# Wirtschaft unter Schock — Finanzpolitik hält dagegen

**DOI:** 10.1007/s10273-020-2629-z

**Published:** 2020-04-22

**Authors:** Oliver Holtemöller, Stefan Kooths, Claus Michelsen, Torsten Schmidt, Timo Wollmershäuser

**Affiliations:** 1 Konjunkturpolitik, DIW Berlin, Mohrenstr. 58, 10117 Berlin, Deutschland; 2 Abteilung Makroökonomik, Institut für Wirtschaftsforschung Halle, Kleine Märkerstraße 8, 06108 Halle (Saale), Deutschland; 3 RWI - Leibniz-Institut für Wirtschaftsforschung, Hohenzollernstraße 1-3, 45128 Essen, Deutschland; 4 Leiter Prognosezentrum, Institut für Weltwirtschaft, Kiellinie 66, 24105 Kiel, Deutschland; 5 und Befragungen, ifo Zentrum für Konjunkturforschung, Poschingerstraße 5, 81679 München, Deutschland

**Keywords:** E32, E66, F01

## Abstract

Nach Ansicht der führenden deutschen Wirtschaftsforschungsinstitute bricht die Konjunktur in Deutschland als Folge der Corona-Pandemie drastisch ein. Um die Infektionswelle abzubremsen, hat der Staat die wirtschaftliche Aktivität hierzulande stark eingeschränkt. Deshalb dürfte das Bruttoinlandsprodukt 2020 um 4,2 % schrumpfen. Die Rezession hinterlässt deutliche Spuren auf dem Arbeitsmarkt und im Staatshaushalt. In der Spitze wird die Arbeitslosenquote auf 5,9 % und die Zahl der Kurzarbeiter auf 2,4 Mio. hochschnellen. Die finanzpolitischen Stabilisierungsmaßnahmen führen 2020 zu einem Rekorddefizit im gesamtstaatlichen Haushalt von 159 Mrd. Euro. Nach dem Shutdown wird sich die Konjunktur schrittweise erholen. Entsprechend fällt der Anstieg des Bruttoinlandsprodukts 2021 mit 5,8 % kräftig aus. Mit dieser Prognose sind erhebliche Abwärtsrisiken verbunden, etwa weil sich die Pandemie deutlich langsamer abschwächen lässt, oder weil das Wiederhochfahren der wirtschaftlichen Aktivität schlechter gelingt als angenommen bzw. eine erneute Ansteckungswelle auslöst.

## Abstract (en)

According to the leading German economic research institutes, the German economy is experiencing a drastic slump as a result of the corona pandemic. In order to slow down the wave of infection, the state has severely restricted economic activity in Germany. As a result, GDP is expected to shrink by 4.2% this year. The recession is leaving clear traces on the labour market and the national budget. At its peak, the unemployment rate will soar to 5.9% and the number of short-time workers to 2.4 million. This year, the fiscal policy stabilisation measures will lead to a record deficit in the general government budget of 159 billion euro. After the shutdown, the economy will gradually recover. Accordingly, the increase in GDP next year will be strong at 5.8%. This forecast is associated with considerable downside risks, e.g. because the pandemic can be slowed faster or because the recovery of economic activity will be less successful than expected or there may be a new wave of infection.

Im Frühjahr 2020 steht die Weltwirtschaft unter dem Eindruck der Corona-Pandemie. Die zuvor erkennbaren Anzeichen für eine Belebung der Konjunktur haben sich im Februar mit der Umsetzung weitreichender Maßnahmen zur Eindämmung der Krankheit in China abrupt umgekehrt. Inzwischen verbreitet sich das Coronavirus weltweit. Nahezu überall werden Maßnahmen ergriffen, um die Ausbreitung zu verlangsamen, die die wirtschaftliche Aktivität massiv bremsen.

Die Corona-Pandemie beeinträchtigt die Wirtschaft über verschiedene Kanäle. Zum einen resultieren Effekte aus einem reduzierten Arbeitsangebot durch höheren Krankenstand und durch krankheitsbedingte Todesfälle. Hinzu kommen seuchenpolitische Maßnahmen sowie Verhaltensänderungen von privaten Haushalten und Unternehmen. Diese beeinträchtigen die wirtschaftliche Aktivität kurzfristig unmittelbar dort, wo viele Menschen auf engem Raum zusammentreffen, etwa durch die Absage von Großveranstaltungen und Messen, und führen zu einem Rückgang im Reiseverkehr. Quarantänemaßnahmen können darüber hinaus die Warenproduktion behindern und die Produktionsabläufe – auch grenzüberschreitend – empfindlich stören. Schließlich drohen Kaufzurückhaltung bei Haushalten und Investitionsattentismus bei Unternehmen, insbesondere weil die Dauer der Einschränkungen der normalen wirtschaftlichen und sozialen Abläufe unsicher ist. Anders als in normalen Rezessionsphasen sind diesmal auch viele Dienstleistungsbereiche stark betroffen, weil sie von den privaten Haushalten entweder freiwillig aus Vorsichtsgründen weniger nachgefragt werden oder ihre Inanspruchnahme durch die Behörden untersagt oder eingeschränkt wird, um die Gefahr von Neuinfektionen zu verringern.

## Maßnahmen zur Eindämmung der Corona-Pandemie und ihrer Folgen

Den negativen Folgen der Ausbreitung des Coronavirus und der Maßnahmen zur Eindämmung der Pandemie ist mit den Instrumenten der Geld- und Finanzpolitik zur Nachfragesteuerung kaum zu begegnen. Die Notenbanken und die Regierungen können aber mit geeigneten Maßnahmen versuchen zu vermeiden, dass ein temporärer Rückgang von Geschäftsvolumen zu dauerhaften realwirtschaftlichen Folgen wie Firmenkonkursen und Arbeitsplatzverlusten führt. So haben viele Notenbanken ihre Leitzinsen gesenkt und massiv zusätzliche Liquidität bereitgestellt. Zielgenauer als die Geldpolitik kann die

Finanzpolitik die realwirtschaftlichen Folgen der Krise abfedern. Hilfen für Unternehmen, Kreditgarantien und einkommensstabilisierende Zahlungen sollen Liquiditätsproblemen bei betroffenen Unternehmen begegnen, um negative Folgen für das Produktionspotenzial so gering wie möglich zu halten. Inzwischen werden in vielen Ländern Haushaltspläne revidiert, um die Wirtschaft zu stützen.

Die Länge und das Ausmaß des globalen Abschwungs hängen vom weiteren Verlauf der Pandemie ab und davon, welche Maßnahmen zu ihrer Verlangsamung ergriffen werden bzw. wie lange diese andauern. Im Zuge der weltweiten Ausbreitung des Coronavirus setzt eine immer größere Zahl von Ländern auf vorbeugende Maßnahmen, welche die wirtschaftliche Aktivität bremsen. Je einschneidender sie sind, desto größer fallen aber kurzfristig die wirtschaftlichen Schäden aus. Unsicher ist zudem, in welchem Umfang auch bei erfolgreicher Verlangsamung der Pandemie seuchenpolitische Maßnahmen notwendig bleiben werden, die die Normalisierung der wirtschaftlichen Abläufe verzögern, solange wirksame Medikamente oder Impfstoffe fehlen. In diesem Gutachten ist unterstellt, dass die Ausbreitung des Coronavirus in den Ländern, in denen die Zahl der Fälle derzeit noch rasch steigt, innerhalb einiger Wochen stark verlangsamt werden kann und sich die globale wirtschaftliche Aktivität spätestens ab Jahresmitte allmählich erholt.

## Globaler Konjunktureinbruch

Ersten Indikatoren zufolge ist die globale Konjunktur massiv eingebrochen. Die Leitindizes an den Börsen sind kräftig gesunken, zeitweise um mehr als 40 %, und die derzeit veröffentlichten Unternehmensbefragungen deuten darauf hin, dass sich die wirtschaftliche Stimmung noch rascher und stärker verschlechtert hat als in der akuten Phase der Weltfinanzkrise. Der Einbruch der wirtschaftlichen Aktivität in China hat für sich genommen bereits im ersten Quartal dazu geführt, dass die globale Wirtschaftsleistung kaum noch gestiegen ist. In der übrigen Welt wird sich die Krise vor allem im zweiten Quartal niederschlagen, sodass die Weltproduktion trotz dann wieder deutlich höherer Aktivität in China im Frühjahr sogar schrumpfen wird. Für die zweite Jahreshälfte rechnen die Institute dann mit einer Erholung der Weltwirtschaft auf breiter Front, sodass die Zuwachsraten der Weltproduktion deutlich höher ausfallen werden als üblich.

Unter diesen Bedingungen unterschreitet die Weltproduktion im Durchschnitt des Jahres 2020 ihr Vorjahresniveau um 2,5 %, ein Rückgang, der noch etwas stärker ist als in der Großen Rezession im Jahr 2009. Für das Jahr 2021 ergibt sich bei einer weitgehenden Annäherung der Produktion an das Niveau, das ohne die Pandemie zu erwarten gewesen wäre, ein Anstieg der globalen Produktion um 5,4 %. Im Vergleich zur Gemeinschaftsdiagnose vom Herbst 2019 bedeutet dies eine Abwärtsrevision um 5,1 Prozentpunkte für das Jahr 2020 und für das Jahr 2021 eine Aufwärtsrevision um 2,7 Prozentpunkte. Der Welthandel dürfte im laufenden Jahr deutlich um 7,4 % einbrechen, im kommenden Jahr dafür mit einer im Vergleich zu den vergangenen Jahren sehr kräftigen Rate von 7 % zunehmen.

## Entwicklungen in Deutschland

Auch die deutsche Konjunktur wird von der Corona-Pandemie voll erfasst und die wirtschaftliche Aktivität stark eingeschränkt. So wurde die Personenmobilität massiv begrenzt, und auf vielerlei Arten von Konsum muss derzeit verzichtet werden. Untersagt wurden insbesondere Dienstleistungen in den Bereichen Freizeit, Unterhaltung, Kultur, Beherbergung und Gaststätten sowie Bildung, Erziehung und Betreuung. Aber auch viele Einzelhändler haben ihre Verkaufsstellen schließen müssen. Unternehmen in anderen Wirtschaftsbereichen sind zwar nicht unmittelbar von diesem staatlich verordneten Shutdown betroffen. Dennoch bekommen sie dessen Folgen zu spüren, auch weil die Menschen

**Prof. Dr. Oliver Holtemöller** ist stellvertretender Präsident und leitet die Abteilung Makroökonomik am Leibniz-Institut für Wirtschaftsforschung Halle.

**Prof. Dr. Stefan Kooths** leitet das Prognosezentrum am Institut für Weltwirtschaft in Kiel.

**Dr. Claus Michelsen** leitet die Abteilung Konjunkturpolitik am Deutschen Institut für Wirtschaftsforschung in Berlin (DIW Berlin).

**Prof. Dr. Torsten Schmidt** ist stellvertretender Leiter des Kompetenzbereichs „Wachstum, Konjunktur, Öffentliche Finanzen“ am RWI Essen.

**Prof. Dr. Timo Wollmershäuser** ist stellvertretender Leiter des ifo Zentrums für Makroökonomik und Befragungen in München.

Tabelle 1
**Eckdaten der Prognose für Deutschland**

| | 2016 | 2017 | 2018 | 2019 | 2020 | 2021 |
|---|---|---|---|---|---|---|
| Reales Bruttoinlandsprodukt (Veränderung gegenüber dem Vorjahr in %) | 2,2 | 2,5 | 1,5 | 0,6 | -4,2 | 5,8 |
| Erwerbstätige im Inland in 1000 Personen | 43.655 | 44.248 | 44.854 | 45.251 | 44.969 | 45.232 |
| Arbeitslose in 1000 Personen | 2.691 | 2.533 | 2.340 | 2.267 | 2.503 | 2.391 |
| Arbeitslosenquote der Bundesagentur für Arbeit[1] in % | 6,1 | 5,7 | 5,2 | 5,0 | 5,5 | 5,3 |
| Verbraucherpreise[2] (Veränderung gegenüber dem Vorjahr in %) | 0,5 | 1,5 | 1,8 | 1,4 | 0,6 | 1,2 |
| Lohnstückkosten[3] (Veränderung gegenüber dem Vorjahr in %) | 1,1 | 1,2 | 2,5 | 3,4 | 3,4 | -0,9 |
| Finanzierungssaldo des Staats[4] | | | | | | |
| in Mrd. Euro | 37,1 | 40,3 | 62,4 | 49,8 | -159,1 | -0,8 |
| in % des nominalen Bruttoinlandsprodukts | 1,2 | 1,2 | 1,9 | 1,4 | -4,7 | 0,0 |
| Leistungsbilanzsaldo | | | | | | |
| in Mrd. Euro | 265,5 | 261,9 | 247,4 | 245,5 | 198,0 | 215,0 |
| in % des nominalen Bruttoinlandsprodukts | 8,5 | 8,1 | 7,4 | 7,1 | 5,9 | 6,0 |

[1] Arbeitslose in % der zivilen Erwerbspersonen (Definition gemäß der Bundesagentur für Arbeit). [2] Verbraucherpreisindex (2010 = 100). [3] Im Inland entstandene Arbeitnehmerentgelte je Arbeitnehmerstunde bezogen auf das reale Bruttoinlandsprodukt je Erwerbstätigenstunde. [4] In der Abgrenzung der Volkswirtschaftlichen Gesamtrechnungen (ESVG 2010).

Quellen: Statistisches Bundesamt; Bundesagentur für Arbeit; Deutsche Bundesbank; 2020 und 2021: Prognose der Institute. © GD Frühjahr 2020.

aus Sorge um ihre Gesundheit von sich aus Konsum reduzieren, wenn er unmittelbaren Kontakt zu anderen Personen mit sich bringt. Auch wird das Arbeitsangebot durch fehlende Kinderbetreuung und Störungen beim grenzüberschreitenden Personenverkehr eingeschränkt. Industrieunternehmen drosselten zudem ihre Produktion teilweise erheblich, da infolge der globalen Pandemie-Bekämpfung Lieferketten gestört und Aufträge weggebrochen sind.

Die Pandemie und die vom Staat ergriffenen Gegenmaßnahmen werden die Konjunktur 2020 und 2021 prägen. Es wurde unterstellt, dass sich das Einfrieren von Teilen der Wirtschaft über eine Dauer von fünf Wochen von Mitte März bis Mitte April erstreckt. Die Abschätzung des daraus resultierenden Ausmaßes der Produktionsbeeinträchtigung in den einzelnen Wirtschaftsbereichen stützt sich auf Verbandsmitteilungen, Medienberichten, Finanzmarktpreisen und die März-Ergebnisse der ifo Konjunkturumfragen. Diese deuten auf eine beispiellose Verschlechterung der Stimmung unter den deutschen Unternehmen in nahezu allen Wirtschaftsbereichen und auf eine massive Zunahme der Unsicherheit hin. Allerdings betrifft der Shutdown die einzelnen Wirtschaftsbereiche unterschiedlich. Aus den branchenspezifischen Schätzungen der Institute resultiert, dass die Wertschöpfung in dieser Phase insgesamt um durchschnittlich etwa ein Fünftel einbricht.

Für den Verlauf der Epidemie wird angenommen, dass das derzeitige Einfrieren des öffentlichen Lebens die Zahl der Neuinfektionen deutlich senkt und deshalb die staatlich verordneten Shutdown-Maßnahmen in der zweiten April-Hälfte allmählich wieder aufgehoben werden. Ferner wird angenommen, dass in der Zeit danach eine effektive Identifikation und Isolation von Infizierten einen schnellen Wiederanstieg der Neuinfektionen verhindern kann. Schließlich wird unterstellt, dass der von der Bundesregierung und den Ländern beschlossene „Schutzschild für Beschäftigte und Unternehmen“ wirkt, dass also Liquiditätshilfen und Zuschüsse sowie das erweiterte Kurzarbeitergeld eine Insolvenzwelle verhindern.

Das Wiederhochfahren der Wirtschaft dürfte sich in den einzelnen Wirtschaftsbereichen in unterschiedlicher Geschwindigkeit vollziehen. Während sich die mit dem Warenkonsum in Verbindung stehende Aktivität unter den getroffenen Annahmen relativ schnell erholen dürfte, wird der Konsum dort, wo er mit sozialen Kontakten in Verbindung steht, wohl noch längere Zeit gedämpft bleiben, auch weil die privaten Haushalte weiter versuchen werden, eine Ansteckung zu vermeiden. Ähnliches dürfte für viele Bereiche des Verarbeitenden Gewerbes gelten, da das Wiederherstellen von Lieferketten Zeit in Anspruch nimmt und die globale Nachfrage insbesondere nach Investitionsgütern zunächst wohl gedämpft bleibt. Weiterhin ist zu berücksichtigen, dass sich infolge des unterstellten Verlaufs der Epidemie Effekte auf das Arbeitsangebot ergeben, da Infizierte (selbst bei mildem Krankheitsverlauf) krankgeschrieben sein werden. Dies dürfte die wirtschaftliche Aktivität insbesondere im Winterhalbjahr 2020/2021 dämpfen, wenn die Infektionszahlen in dem in dieser Prognose unterstellten Szenario ihren Höhepunkt erreichen.

Unter diesen Annahmen schrumpft das Bruttoinlandsprodukt bereits im ersten Quartal 2020 um 1,9 % und bricht im zweiten Quartal um 9,8 % ein. Dies ist der stärkste je seit Beginn der Vierteljahresrechnung im Jahr 1970 gemessene Rückgang in Deutschland und mehr als doppelt so groß wie jener während der Weltfinanzkrise im ersten Quartal 2009. Der Einbruch bei den Ein- und Ausfuhren, den Ausrüstungsinvestitionen und dem privaten Konsum dürfte noch stärker ausfallen und im zweistelligen Bereich liegen. Nach dem Shutdown wird sich die Konjunktur schrittweise erholen. Ein Großteil der Ausfälle beim privaten Konsum, bei den Investitionen und beim Export wird rasch wieder aufgeholt, was sich in hohen Zuwachsraten niederschlägt. Das Vorkrisenniveau wird aber wohl erst gegen Ende des Prognosezeitraums wieder erreicht. Im Durchschnitt des Jahres 2020 dürfte der Rückgang der gesamtwirtschaftlichen Produktion 4,2 % betragen (vgl. Tabelle 1). Dies wäre nach 2009 die tiefste Rezession seit dem Ende des Zweiten Weltkriegs; allerdings fällt der Rückgang nicht so drastisch aus wie zu Zeiten der großen Depression. Im Vergleich zum Herbst 2019 revidieren die Institute ihre Prognose für das laufende Jahr kräftig um 5,3 Prozentpunkte nach unten. Im kommenden Jahr schlägt die konjunkturelle Erholung zu Buche: Der Anstieg des Bruttoinlandsprodukts fällt mit 5,8 % kräftig aus.

## Folgen für den Arbeitsmarkt, die verfügbaren Einkommen und den Staatshaushalt

Die Corona-Krise hat spürbare Auswirkungen auf den Arbeitsmarkt. Infolge des Einbruchs der gesamtwirtschaftlichen Aktivität fällt das Arbeitsvolumen kurzfristig beträchtlich. Dabei dürfte die Erwerbstätigkeit in diesem Jahr um 282.000 Personen zurückgehen und die Zahl der Arbeitslosen um 236.000 steigen. Die Arbeitslosenquote wird auf 5,5 % der Erwerbspersonen zunehmen. Ihren Höchststand dürfte die Arbeitslosigkeit mit rund 2,7 Mio. Personen bzw. einer Quote von 5,9 % im dritten Quartal 2020 erreichen. Der größte Teil der Anpassung wird jedoch über einen kräftigen Rückgang der Arbeitsstunden je Erwerbstätigen erfolgen. Hier schlagen sich auch die von der Bundesregierung beschlossenen Maßnahmen zur Beschäftigungssicherung nieder, mit denen insbesondere die Zugangsvoraussetzungen zum Kurzarbeitergeld deutlich gelockert wurden. Somit dürfte die Zahl der Kurzarbeiter von schätzungsweise 110.000 Personen Anfang 2020 auf etwa 2,4 Mio. im Durchschnitt des zweiten Quartals hochschnellen.

Die verfügbaren Einkommen der privaten Haushalte werden im laufenden Jahr erstmals seit der Großen Rezession sinken. Sowohl die Lohnsumme als auch die Einkommen aus selbständiger Arbeit und Vermögen werden zurückgehen. Dem wirkt der beschleunigte Anstieg der monetären Sozialleistungen infolge steigender Kurzarbeit und Arbeitslosigkeit entgegen. Gestützt wird die Kaufkraft der privaten Haushalte 2020 durch den geringeren Preisauftrieb, der vor allem aus dem Absturz der Rohölpreise resultiert. Die Verbraucherpreise werden in diesem Jahr nur um 0,6 % steigen.

Die Corona-Krise hinterlässt auch deutliche Spuren im Staatshaushalt. Die öffentlichen Einnahmen aus Steuern und Sozialbeiträgen werden sich im laufenden Jahr aufgrund des konjunkturellen Einbruchs und der von Bund und Ländern verabschiedeten steuerlichen Maßnahmen zur Eindämmung der Folgen des Shutdowns deutlich verringern. Auch die Entwicklung der Ausgaben des Staats wird 2020 maßgeblich durch die finanzpolitischen Stabilisierungsmaßnahmen im Zuge der Corona-Krise beeinflusst. Darin schlagen sich unter anderem die Ausweitungen des Kurzarbeitergelds, Zuschüsse an Unternehmen und Beschaffungen im Gesundheitswesen nieder. Die hohen Mehrausgaben und Mindereinnahmen führen in diesem Jahr zu einem Rekorddefizit im gesamtstaatlichen Haushalt von 159 Mrd. Euro. Im Jahr 2021 dürfte der Staatshaushalt dann wieder in etwa ausgeglichen sein. Der Bruttoschuldenstand des Staates in Relation zum nominalen Bruttoinlandsprodukt wird unter Berücksichtigung des hohen finanziellen Defizits und der damit verbundenen Neuverschuldung, sowie der zusätzlichen Kredite und Beteiligungen von 250 Mrd. Euro in diesem Jahr auf etwa 70 % steigen. Im Jahr 2021 dürfte er dann – vor allem aufgrund des wieder höheren nominalen Bruttoinlandsprodukts – auf rund 64 % sinken.

Insgesamt bewirkt die Corona-Pandemie somit eine schwerwiegende Rezession in Deutschland. Die Beeinträchtigungen durch die Pandemie selbst dürften jedoch nach ein bis zwei Jahren überwunden sein. Deutschland bringt gute Voraussetzungen mit, den wirtschaftlichen Einbruch zu verkraften und mittelfristig wieder das wirtschaftliche Niveau, das sich ohne die Krise ergeben hätte, zu erreichen. Die günstige fiskalische Ausgangssituation ermöglicht es dem Staat, weitgehende Maßnahmen zur Abfederung der kurzfristigen negativen Folgen für Unternehmen und private Haushalte zu ergreifen. Dennoch bleiben gesamtwirtschaftliche Einbußen, die mit individuell sehr unterschiedlichen Lasten verbunden sind und über deren finale Verteilung noch zu entscheiden sein wird.

## Prognoserisiken

Mit dieser Prognose sind erhebliche Abwärtsrisiken verbunden. So ist es nicht unwahrscheinlich, dass sich die Krise länger hinzieht, etwa weil sich die Pandemie deutlich langsamer abschwächen lässt, oder weil das Wiederhochfahren der wirtschaftlichen Aktivität schlechter funktioniert als im Basisszenario bzw. eine erneute Ansteckungswelle auslöst. Auch können weitere Maßnahmen zur Infektionsbekämpfung in Kraft treten, die die Produktion länger oder in größerem Umfang als hier angenommen stilllegen. Solche Produktionsstillstände ziehen kurzfristig erhebliche gesamtwirtschaftliche Effekte nach sich. Diese dürften mit zunehmender Dauer überproportional steigen. Ein solches Szenario verstärkt den Konjunktureinbruch, verlängert die Rezession und verlangsamt die Erholung. Auch werden dann Verwerfungen im Finanzsystem als Folge vermehrter Unternehmensinsolvenzen wahrscheinlicher, die durch staatliche Schutzschilde nicht verhindert werden können.

Schließlich durchlaufen auch die anderen Länder des Euroraums tiefe Rezessionen, die die Staatsfinanzen unter Druck setzen. Angesichts der zum Teil bereits erheblichen Staatsschuldenstände dürften mit der Dauer und Tiefe des Einbruchs in einigen Fällen Zweifel an der Schuldentragfähigkeit steigen. Sollte es zu einer zweiten Welle von Staatsschuldenkrisen im Euroraum kommen, würde dies die Absatzaussichten deutscher Exporteure und die Finanzstabilität im Euroraum insgesamt zusätzlich belasten. Wenn es in anderen Staaten zu Insolvenzwellen kommt, dürfte dies die Produktionsstrukturen erheblich beschädigen. Eine damit verbundene Neuausrichtung globaler Wertschöpfungsketten und Absatzmärkte wäre für das deutsche Verarbeitende Gewerbe mit deutlich höheren Kosten verbunden als hier unterstellt.

Zu den wenigen Aufwärtsrisiken, die zu einer besseren als der hier prognostizierten konjunkturellen Entwicklung führen würden, zählt insbesondere ein günstigerer Verlauf der Pandemie. So könnte nach dem Shutdown die Zahl der Neuinfektionen schneller und stärker sinken, etwa weil schnelle Fortschritte bei der Entwicklung eines Impfstoffes erzielt werden können bzw. sich im Zeitablauf die Immunität der Bevölkerung generell als höher erweist oder sich der saisonale Einfluss auf die Ausbreitung des Coronavirus als größer herausstellt. Zudem könnte vor diesem Hintergrund die konjunkturelle Erholung nicht nur in Deutschland, sondern auch bei den deutschen Handelspartnern, zügiger vonstattengehen.
